# The Microstructure Evolution and Formation Mechanism of Gradient Nanostructure Prepared on CrCoNi Medium-Entropy Alloy

**DOI:** 10.3390/nano13131954

**Published:** 2023-06-27

**Authors:** Dou Ning, Wenjie Lu, Xian Luo, Yanqing Yang, Bin Huang

**Affiliations:** 1 State Key Laboratory of Solidification Processing, Northwestern Polytechnical University, Xi’an 710072, China; nd5635265@163.com (D.N.); luwenjie@nwpu.edu.cn (W.L.); yqyang@nwpu.edu.cn (Y.Y.); huangbin@nwpu.edu.cn (B.H.); 2 Langu Institute for Materials Analysis Co., Ltd., Weihai 264207, China

**Keywords:** medium-entropy alloys, plastic deformation, gradient nanostructure, microstructure evolution, grain refinement

## Abstract

An equiatomic CrCoNi medium-entropy alloy was subjected to high-energy shot peening (HESP) to fabricate a gradient nanostructure (GNS) in this work. The microstructures of the GNS samples at different depths within the deformed layer were thoroughly investigated. The microstructure exhibited a transformation from unstressed coarse grains to deformed coarse grains, followed by the formation of ultrafine grains, and ultimately reaching a final nanocrystalline structure with a uniform size of approximately 50 nm. Detailed structural analysis indicated that the deformation process was primarily influenced by the interaction between dislocations and deformation twins, which was attributed to the low stacking fault energy (SFE) of the alloy. The nanocrystalline mechanism was divided into three stages. In the coarse-grained deformation stage, the dislocation band divided twin/matrix lamellae into sub-segments, and the cross twin divided coarse grains into ultrafine grains simultaneously. In the ultrafine grain deformation stage, dislocations were arranged around the deformation twins in order to break the twins to form incoherent boundaries, destroying the coherent relationship between the twin and matrix. Finally, in the nanocrystalline deformation stage, the nanocrystalline structure was further divided into smaller segments to accommodate additional strains through the interaction between dislocations and twins.

## 1. Introduction

Medium/high-entropy alloys (MEAs/HEAs) have sparked the interest of academics, due to their excellent properties achieved through their unique composition design, since their discovery in 2004 [1,2,3,4,5]. Extensive research with the FCC structure type has primarily focused on FeCoNiCrMn HEA and other alloys with ternary, quaternary, and even more than six elements obtained through composition changes on this basis [6,7,8]. The ternary alloy CrCoNi MEA always exhibits higher strength and plasticity, especially at ambient and low temperatures, owing to its stable solid solution structure and low staking fault energy (SFE) of (22 ± 4) mJ/m^2^ [9,10]. Excellent alloy design is reflected not only in the composition design [11,12], but also in the structure design. The design of heterogeneous structures through heat treatment or severe plastic deformation may significantly affect the mechanical behavior of materials [13,14,15].

Recently, researchers have primarily used severe plastic deformation methods, such as high-pressure torsion [16,17], hard turning [18,19], and ultrasonic surface modification [20,21], to prepare the gradient structures of MEAs/HEAs in order to create a synergy between the related stress/strain gradient and the microstructure gradient on the surface. For example, Guo et al. prepared a gradient structure on the surface of CrCoNi MEA through hard turning. A hierarchical structure was formed in the nanocrystals, including martensite, twin, and FCC phases [18]. Chen et al. reported a gradient structural Al_0.1_CoCrFeNi HEA treated by cyclic dynamic torsion. The crystal defects experienced an evolution from dislocation lines to high-density dislocation walls and dislocation cells, and then to nanotwins caused by the stress gradient. The gradient structure could produce a synergistic enhancement effect on materials [16].

Until now, investigations of these nanocrystallization mechanisms have primarily focused on conventional metallic materials, such as Cu [22], Ni [23,24], 304 stainless steel [25], and so on. The material deformation mechanism occurring through severe plastic deformation is related to the material crystal structure and SFEs [26]. The SFEs are critical in determining the plastic deformation modes of materials in FCC metals. The deformation of high-SFE materials is mainly driven by dislocation activity, which includes dislocation multiplication, accumulation, rearrangement, and annihilation. Dislocation activity can generate dislocation cells, walls, or tangles through slip to subdivide grains [27,28,29,30]. The dislocation–twin interaction and twin–twin interaction are the main routes to achieve grain refinement in moderate- and low-SFE metals [19,31,32,33].

However, due to severe lattice distortion, the deformation mechanism of equiatomic multi-component alloys may differ from that of traditional alloys. The grain refinement mechanism of FeCoCrNi HEA prepared by HPT was studied. The nanocrystalline grains were mainly formed via concurrent nanoband subdivision and deformation twinning [34]. In this study, a CrCoNi MEA with low SFE was chosen and processed by HESP to create a gradient nanostructure layer on the sample. The plastic strain gradient and grain size gradient were characterized using electron backscattering diffraction (EBSD) and transmission electron microscopy (TEM) at the depth of 0~250 μm. TEM observations were used to systematically study the detailed microstructure and nanocrystallization mechanism of the nanograins. The results showed that the deformation process was closely related to the evolution and interaction of the dislocations and deformation twins.

## 2. Experimental Procedures

### 2.1. Sample Preparation

In this work, the button-shaped ingot of an equiatomic mixture of high-purity (99.97 wt%) Cr, Co, and Ni was melted in a non-consumable vacuum arc melting furnace with a Ti-gettered high-purity argon atmosphere. The ingot was flipped and re-melted at least five times, and then homogenization annealing treatment was performed at 1200 °C for 12 h, followed by water quenching. Then, the ingot was cold-rolled with a thickness reduction of 10 to 2 mm (80% reduction). Rectangular-shaped samples of 50 × 20 × 2 mm^3^ in dimension were cut using electrical discharge machining. Then, the cut pieces were kept at 700 °C for 1 h for recrystallization annealing, and then cooled to room temperature with water.

**Figure 1:**
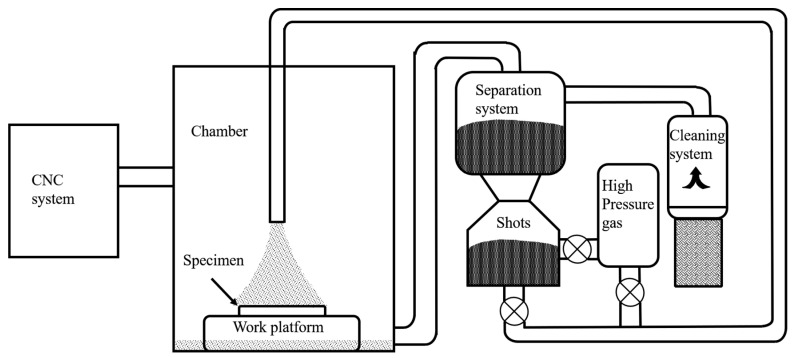
Schematic illustration of the CNC shot peening system.

The sample was mechanically polished using silicon carbide abrasive paper until 2000# before the shot peening treatment. High-energy shot peening (HESP) was conducted on both sides of the sheet using a CNC LSSKPWJ1512P-15 shot peening machine (seen in Figure 1). The material of the shots was ASH 230 cast steel with a diameter of 0.6 mm. During the shot peening process, the distance between the spray gun and specimen was 300 mm and the spray gun angle was 90°. Regarding the shot parameters, an air pressure of 0.35 MPa and a duration of 20 min were set in this work.

### 2.2. Microstructural Characterization

The variation in microhardness on the cross-sections of gradient samples was measured at a load of 0.98 g for 15 s; measurements of the samples were repeated at 5 independent positions. The longitudinal section microstructure of the gradient nanostructured (GNSed) CrCoNi MEA sample was characterized by EBSD using a Zeiss SIGMA500 field-emission scanning electron microscope (SEM) equipped with an Oxford Nordlys Max3 EBSD detector. The EBSD and SEM specimens were prepared by mechanical polishing and then electrolytic polishing using a solution of 10% perchloric acid and 90% ethanol with a direct voltage of 10 V for 8~12 s. Detailed microstructure observation was conducted by TEM and high-resolution TEM (HRTEM) on a Talos F200s transmission electron microscope operated at a voltage of 200 kV. Prior to the TEM observation, the slices were cut and mechanically ground to 70 μm, and then thinned with an ion beam. The topmost TEM analysis specimen was prepared by focused ion beam (FIB) milling using a TESCAN LYRA3 GMH FIB-SEM dual-beam system.

## 3. Results and Discussion

### 3.1. Overviews of GNSed CrCoNi MEA

#### 3.1.1. The Hardness of GNSed CrCoNi MEA

**Figure 2:**
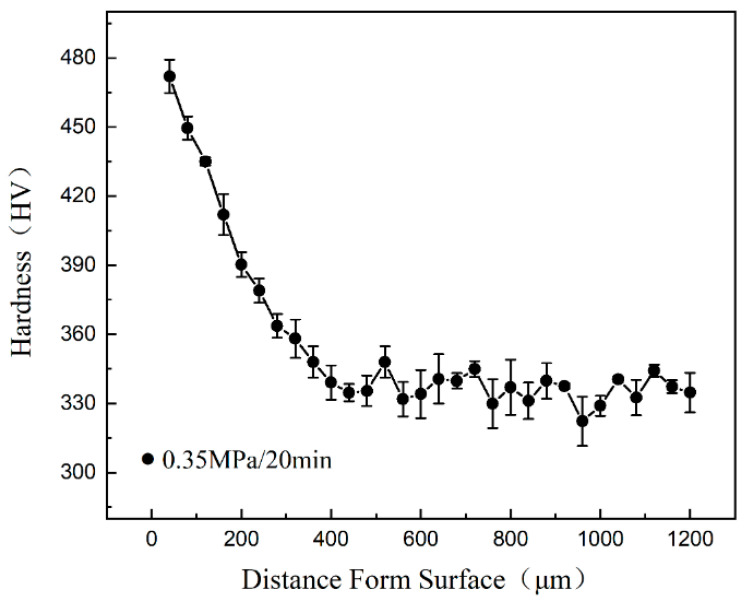
The hardness of the GNSed CrCoNi MEA processed by HESP at an air pressure of 0.35 MPa for 20 min.

The hardness gradient on the cross-section of the sample after HESP is presented in Figure 2. The topmost surface layer had a hardness value of (472 ± 7) HV, which gradually dropped to (335 ± 6) HV at the depth of approximately 0.25 mm. It can be seen that the surface hardness of the sample was significantly improved (approximately 45% increment) after HESP. This observation indicates the presence of a gradual microstructural transition, where the severely deformed fine grains on the surface gradually transition to recrystallized grains in the center of the material.

#### 3.1.2. The Microstructure of GNSed CrCoNi MEA

**Figure 3:**
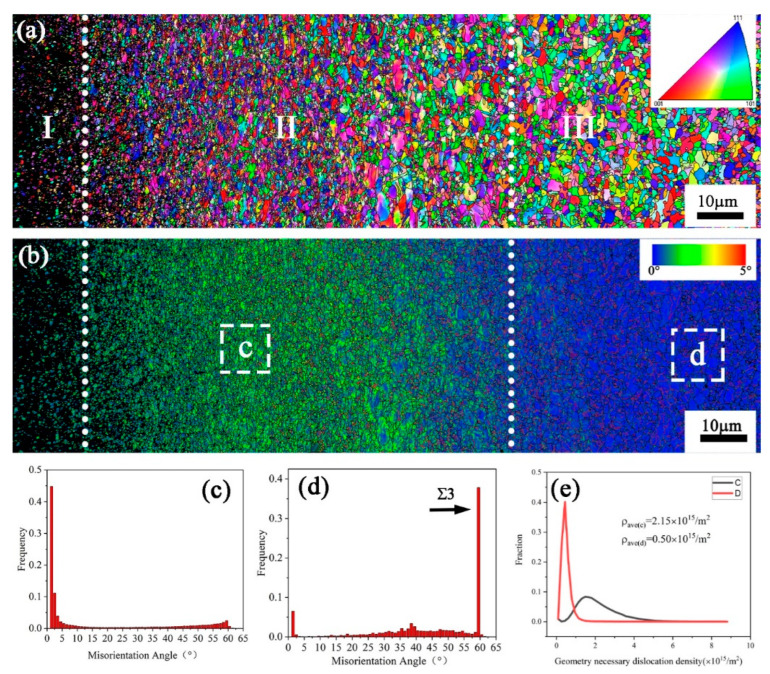
EBSD images of the GNS CrCoNi MEA processed by HESP at an air pressure of 0.35 MPa for 20 min from depth of 40 μm to 230 μm: (**a**) grain orientation map; (**b**) local misorientation map; (**c**,**d**) grain boundary misorientation maps corresponding to the white frames c and d in (**b**), respectively; (**e**) calculated GND density of the white frames c and d in (**b**), respectively.

Figure 3 shows the GNSed CrCoNi MEA fabricated by HESP and characterized using EBSD. Based on the grain size, the gradient nanostructure can be categorized into three regions, the high-stress layer, the medium-stress layer, and the low-stress layer, corresponding to the nanocrystalline zone, ultrafine grain zone, and coarse grain zone, respectively. In Figure 3a, the coarse grains are observed to be broken into equiaxed grains with a gradient scale from the surface to core under the random action of shots with a random grain orientation. The local misorientation map in Figure 3b clearly indicates the variation in plastic deformation from the surface to the core, induced by HESP. The plastic deformation is most pronounced near the surface layer, and, as the depth increases, the level of plastic deformation gradually decreases, with almost no deformation observed in the core region. The grain boundary misorientation of the C and D zones in Figure 3b is significantly different, as seen in Figure 3c,d. In Figure 3c, a considerable number of non-equilibrium grain boundaries and low-angle grain boundaries (LAGBs) with misorientation angles of less than 10° are observed near the shot-peened surface layer, accounting for approximately 70.30% of the total grain boundaries in zone C. In contrast, the proportion of such boundaries in zone D is only 9.14%. The black arrow in Figure 3d indicates that there is high content of grains with a 60° misorientation, representing a high density of annealed twins, which is attributed to the low SFE of the CrCoNi MEA, which is only (22 ± 4) mJ/m^2^ [10].

The geometrically necessary dislocation (GND) density can be calculated to describe local strain using KAM-EBSD qualitatively. The misorientation angle θ is related to the GND density ρ_GND_, which has the following relationship [35,36]:

(1) $$\rho_{GND}=\frac{2\theta }{ub}$$

where *u* is the unit scanning step of EBSD and *b* is the Burgers vector. The values of *u* and *b* are 8 × 10^−8^ m and 2.5 × 10^−10^ m, respectively.

Figure 3e shows the comparison of the GND density between the C and D zones, and it is observed that the deformed structure with higher stress accumulation results in a higher density of dislocation accumulation. The calculated average densities are 2.15 × 10^15^ m^−2^ and 0.50 × 10^15^ m^−2^ in the C and D zones, respectively, indicating the presence of a significant plastic strain gradient. The increase in GND density indicates an enhanced level of lattice distortion. This distortion within the crystal lattice facilitates the movement and migration of grain boundaries, leading to the ultimate refinement of grains [37,38].

**Figure 4:**
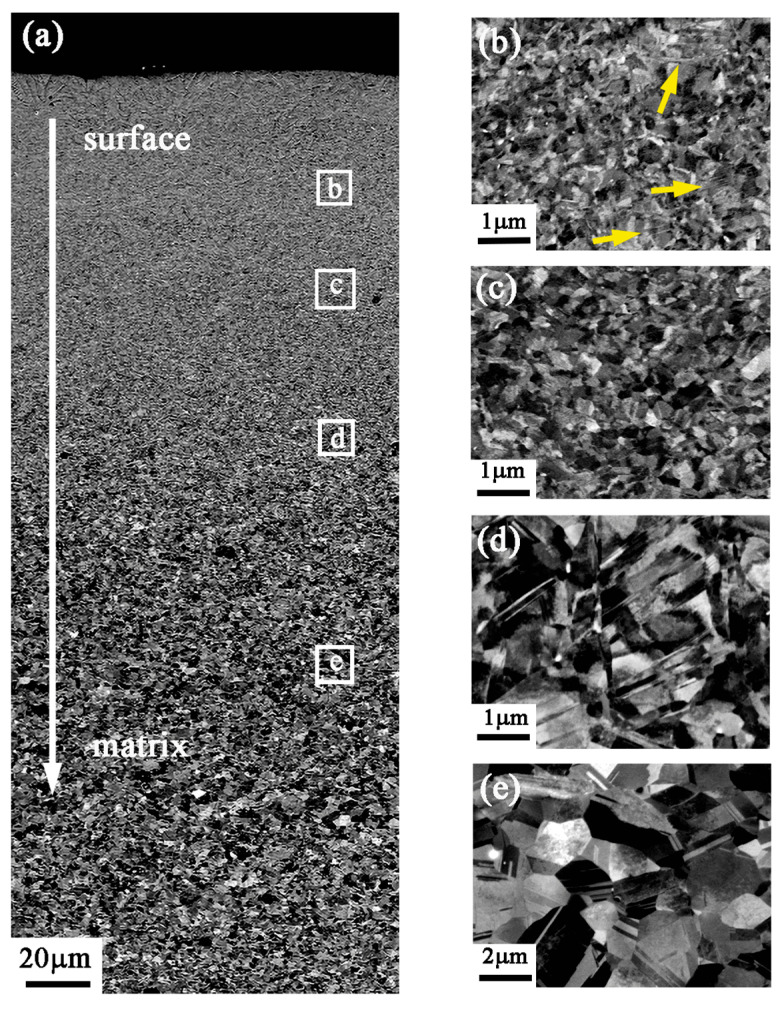
SEM images of the cross-sectional microstructure of the GNS CrCoNi MEA after HESP treatment: (**a**) full view of the gradient deformation layer; (**b**–**e**) enlarged views at 40, 70, 120, and 200 μm depth from surface, corresponding to the white frames b, c, d, and e in (**a**).

The gradient structure along the depth from the treated surface to the matrix was also characterized by SEM–electron channeling contrast imaging (ECCI), as is shown in Figure 4. In Figure 4a, the near-surface layer exhibits a pronounced gradient structure without a clear boundary. The strain varies at different depths, resulting in different microstructures. Approximately 40 μm away from the treated surface, the observed deformation microstructure shows the significant presence of nanometer-scale deformation twins, as indicated by the yellow arrow. The grains are seriously deformed and the grain boundaries are indistinguishable under a high stress level. The observed grain scale falls within the ultrafine range, and the grains undergo refinement from ultrafine to nanocrystalline, as depicted in Figure 4b. Figure 4c presents the captured grain information at a depth of approximately 70 μm, where the coarse grains have been refined to ultrafine grains. The clear contrast indicates that the ultrafine and equiaxed grains in this area can be identified. The refinement process from a coarse grain to a fine grain structure is shown in Figure 4d. This region is approximately 120 μm from the surface, showing a typical deformed structure. The contrast variation in reflection in the SEM image suggests the likely presence of a high density of dislocations in the grains. Dislocations are seen around deformed twins and grain boundaries, making it difficult to resolve the boundaries of coarse grains. Figure 4e shows a fully annealed structure with distinct grain boundaries and numerous annealing twins at a depth of 200 μm.

**Figure 5:**
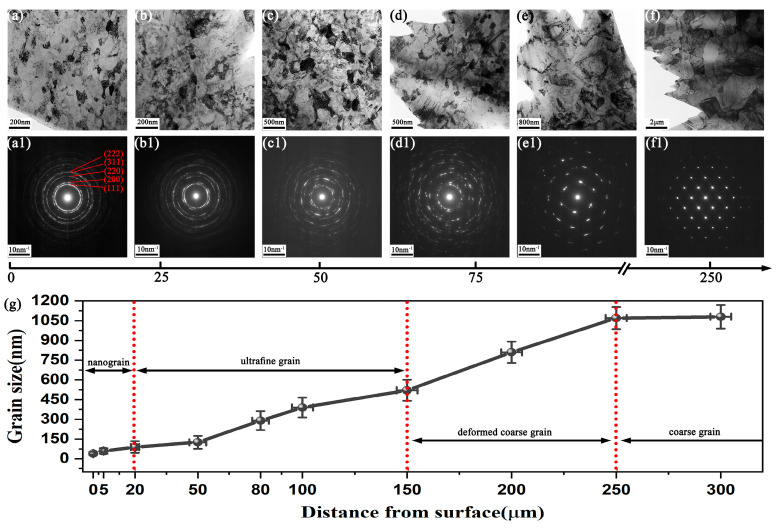
The microstructure evolution and grain size variation at different depths from the surface after HESP: (**a**–**f**) TEM images of grain morphology at 5, 20, 50, 80, 150, and 250 μm depth from the surface, respectively; (**a1**–**f1**) the SAED patterns corresponding to (**a**–**f**), respectively; (**g**) the grain size distribution curve from surface to core.

A series of representative microstructure evolutions at different depths from the surface were characterized by TEM, and an aperture with a diameter of 800 nm was used to obtain the electron diffraction patterns, as shown in Figure 5a–f and a1–f1. In the topmost layer, equiaxed grains with an average size of approximately 50 nm are uniformly distributed, and the polycrystalline diffraction circle appears continuous, indicating the presence of numerous nanograins with random orientations. The diffraction pattern corresponds to the five diffraction planes, (111), (200), (220), (311), and (222), of the FCC phase. As the depth increases from the surface, the grain size gradually increases and the diffraction rings gradually become discontinuous. The average grain sizes at depths of 20 μm, 50 μm, and 80 μm increase to approximately 90 nm, 125 nm, and 290 nm, respectively. At a depth of 150 μm, a prominent microstructural feature is the entanglement of dislocations around the grains, and deformation twins embedded with high-density dislocations can be seen in Figure 5d. The diffraction pattern demonstrates that there are only one or two grains in the selected area. At a further depth of 250 μm, the microstructure comprises coarse grains with distinct grain boundaries and low-density dislocation lines, and the average grain size is approximately ~1.1 μm. The SAED pattern with a set of regular diffraction spots indicates that there is no strain in this area. It is noted that the grains always remain equiaxed from the surface to the core, indicating the random force direction loaded on the sample. The variation in the average grain size along the depth is presented in Figure 5g. Based on the grain size, it can be concluded that the surface gradient nanostructure is composed of four regions: a nanograin layer (0~20 μm), an ultrafine grain layer (20~150 μm), a deformed coarse grain layer (150~250 μm), and an unstressed coarse grain layer (>250 μm).

### 3.2. Detailed Structure of Different Layers on the Sample Surface

In this section, the evolution of dislocations and deformation twins and their contributions to grain refinement are further explored through a detailed analysis of the internal characteristics of grains at different layers. The three stages of grain refinement, i.e., coarse grain deformation stage, ultra-fine grain deformation stage, and nanocrystalline deformation stage, are analyzed.

**Figure 6:**
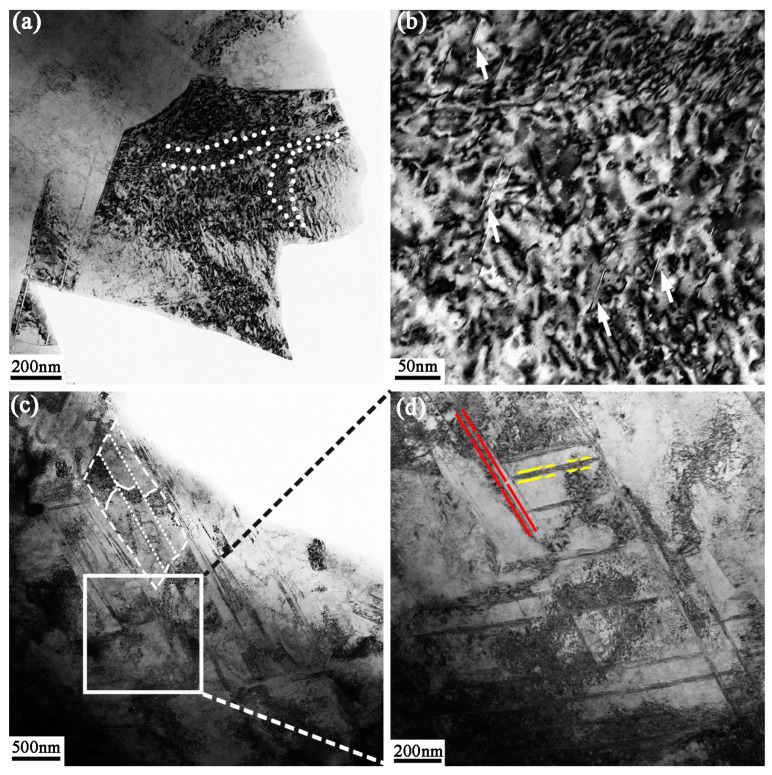
(**a**) TEM image of microstructure at 200 μm depth; (**b**) an enlarged BF image of dislocations and stacking faults in (**a**); (**c**) TEM image of deformation twins at 150 μm depth; (**d**) an enlarged BF image corresponding to the white frame in (**a**).

The feature of dislocation and deformed twins in the coarse grain deformation stage is presented in Figure 6. Figure 6a shows a grain that still remains equiaxed, and there are high-density dislocation structures in the grain compared to the grains in Figure 5e. The short and dense dislocation lines shown by the white dashed lines are arranged inside the grain to separate the grain. In addition, Figure 6b shows the presence of numerous dislocation entanglements and short rod-like stacking faults shown by the white narrows within the grain. This indicates that dislocation activity is the predominant mechanism during the initial stages of plastic deformation. The refinement of coarse grains is more likely to be dominated by the interaction between twins and dislocations with a much higher strain gradient. A view of the twin/matrix lamellae can be seen when the stress level is gradually increased at 150 μm depth in Figure 6c. The dislocation bands break up the twin/matrix lamellae through accumulation and interaction, as shown by the white dashed frame. In addition, cross twins separate the grains into rhomboid blocks with high misorientation noticeably, as indicated by the yellow and red dashed lines in Figure 6d.

**Figure 7:**
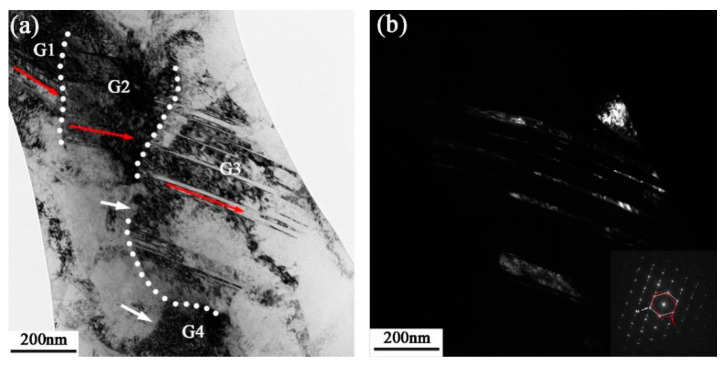
TEM image of microstructural morphologies at 80 μm depth; (**a**) BF image; (**b**) DF image and corresponding SAED pattern.

In comparison with the segmentation phenomenon at 150 μm depth, the coarse grains in Figure 7 have been refined to ultrafine grains at 80 μm depth. The red arrows in Figure 7a indicate that there are deformation twins in G1, G2, and G3 grains with different orientations. It should be noted that the high-density dislocations surround and interact with the twins, resulting in the twins gradually deviating from their twinning relationship with the matrix. As shown by the white arrow in G3, the dislocation lines are arranged closely in the G3 boundary to increase misorientation and break up the twins. The dislocation bands indicated by the white arrow in G4 divide the grain into smaller sizes.

**Figure 8:**
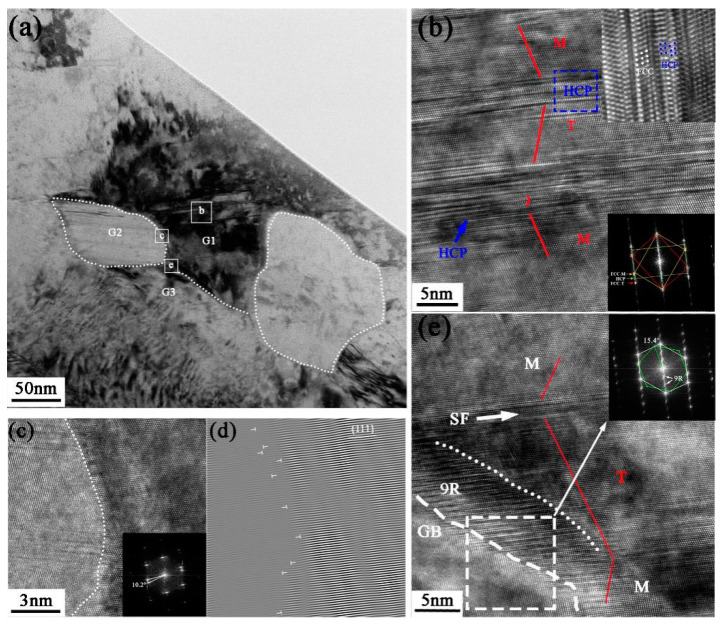
(**a**) TEM image of detailed microstructural morphology at 50 μm depth; (**b**) HRTEM image of deformed twin corresponding to the white frame b in (**a**); (**c**) HRTEM image and FFT image of sub-grain boundary corresponding to the white frame c in (**a**); (**d**) IFFT image corresponding to (**c**); (**e**) HRTEM image of grain boundary corresponding to the white frame e in (**a**).

The detailed microstructure at 50 μm depth from the surface is shown in Figure 8a, in which the ultrafine grains can be observed undergoing further deformation and refinement, resulting in even finer structures. There is a significant difference in diffraction contrast between these grains, indicating that the grains are randomly oriented, and a nanograin G2 (the left grain in Figure 8a) with a grain size of approximately 79 nm is formed at the intersection region. The HRTEM image of the deformation twin in G1 is shown in Figure 8b, which is broken by the slipping of dislocations in G2 boundaries. High-density dislocation and a stacking fault structure are observed with the twin boundaries, resulting in an extremely complex twin boundary structure. With the accumulation of dislocations at the twin boundary, the {111} planes originally parallel on both sides of the TB are no longer parallel to each other. A deformation nanotwin with three atomic planes was also observed as a result of the continuous slipping of Shockley dislocations at the plane (111) [39]. Notably, the HCP laths are formed in nanotwins, resulting in a nanotwin–HCP lamella structure in the blue frame (seen in Figure 8b). The HCP laths have a consistent orientation relationship with the FCC matrix, which are {111}_FCC_∥{0001}_HCP_ and [110] _FCC_∥[1120]_HCP_ [40], and this relationship is supported by the FFT analysis shown in the insert in Figure 8b. The slip of Shockley partial dislocation with Burger vector a/6<112> generates intrinsic stacking faults, which subsequently nucleate and expand on the adjacent (111) slip planes to form local HCP stacking [41]. The phenomenon of stress-induced phase transformation is commonly seen in high-Mn austenitic steels [42,43]. Recently, the phase transition from an FCC structure to an HCP structure has also been found in MEAs with high strain or lower stacking fault energy, such as Co_27_Cr_5_Mo_0.05_C [44], Fe_20_Mn_20_Ni_20_Co_20_Cr_20_ [45], and NiCrCo [40,46] MEAs. Figure 8c,d show the atomic-filtered images to dissect the atomic arrangement and crystalline defects. From the analysis of a one-dimensional IFFT image, it is evident that there is a high concentration of dislocations at the grain boundaries, whereas the dislocation density inside the grains is relatively low. This observation suggests that the rotation of sub-grains is induced by dislocation slip, resulting in increased misorientation between adjacent grains and ultimately leading to the formation of high-angle grain boundaries (HAGBs). Figure 8e gives the HRTEM image of the atomic information of the interface between G1 and G3, which has a misorientation of ~15.4° with respect to each other according to the FFT image. A typical 9R phase structure is observed along the twin boundary, as evidenced by the additional diffraction points with three plane spacings in the SAED pattern inserted in Figure 8e. It has been reported that the 9R structure always exists as the nucleation point of deformation twins in the grain, and the deformation twins generally nucleate at grain boundaries [47]. In addition, stacking faults can be found along the twin boundaries.

**Figure 9:**
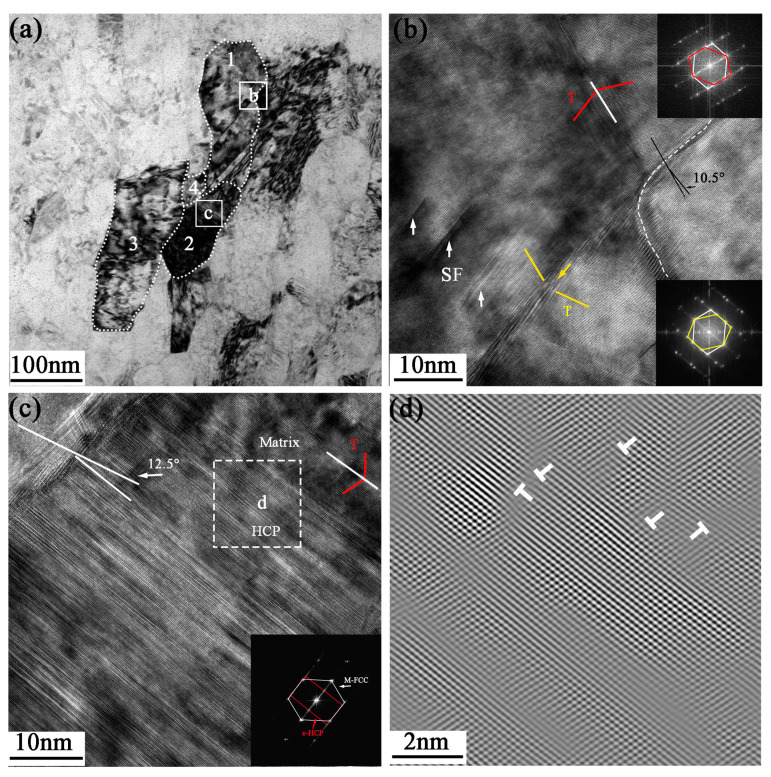
(**a**) TEM image of the grains at 5 μm depth; (**b**) HRTEM image of deformed twins corresponding to the white frame b in (**a**) and FFT images of twin grain boundaries; (**c**) HRTEM image of grain corresponding to the white frame c in (**a**) and FFT image; (**d**) IFFT image corresponding to the white frame d in (**c**).

Figure 9a depicts the grain segmentation characteristics at a distance of approximately 5 μm from the surface, where the grains have been essentially refined to the nanometer scale. The grains appear elongated, with short-axis lengths of less than 100 nm. Deformation twins in opposite directions are produced as a result of severe plastic deformation in G1 (The numbers 1, 2, 3, and 4 represent the G1, G2, G3, and G4 grains, respectively). Step-shaped twins are formed in the lower region of G1, and the twin/matrix lamellae are disrupted at the boundaries of G2 and G4 by dislocation activity. Under high stress, dislocations gradually divide the twins and form sub-grain boundaries with random orientations. The detailed structure in G1 is shown in Figure 9b, in which the red line specifically shows the symmetrical relationship between the twin and the matrix around the axis [011]. The upper FFT image confirms that the twin plane is (11$\bar{1}$), while the yellow line indicates that the twin plane is (1$\bar{1}$1). There is a complex atomic arrangement at the twin boundary, and the presence of dislocations and stacking faults leads to the formation of several steps on the atomic plane. When two twins intersect, the grain rapidly separates into smaller sub-structures. Stacking faults are indicated by the white arrows, and Shockley partial dislocations can be found nearby. A HAGB with a misorientation angle of approximately 10.5° is formed between G1 and the adjacent grain. G4 is a nanograin formed by the interaction between dislocations and twins. The misorientation angle is approximately 12.5° between G3 and G4 according to the atomic information in Figure 9c. The grain exhibits a high density of stacking faults and micro-twin structures, and the slip of stacking faults under part of the grain results in a multi-layer HCP structure in the atomic arrangement, as evidenced by the extra diffraction spots of ε-HCP in the FFT image. It should be noted that partial dislocations and lattice distortion exist at the interface between the HCP and FCC matrix, as depicted in Figure 9d.

### 3.3. Deformation Mechanism

The gradient nanostructure layer was prepared on the surface of CrCoNi MEA after HESP. The high density of dislocations and twins always exists in the deformed grains, new boundaries are constantly formed in the deformation process, and segmented microstructures appear on smaller scales. The deformation mechanism, the interactions between twins and dislocations, and the grain refinement mechanism can be summarized in the process of microstructure evolution.

#### 3.3.1. Dislocation and Twin Evolution

The current study discovered that as the stress level increases, the evolution of dislocations in the grains progresses from low-density dislocation lines to high-density dislocation entanglement and eventually to much higher-density dislocation bands. However, after the formation of a nanocrystalline structure, the dislocation density in the grains gradually decreases. Dislocation activity always plays a crucial role during the deformation process. Firstly, the slip of Shockley partial dislocation can generate stacking faults and deformation twins, which is a typical phenomenon in FCC metals with low SFE during severe plastic deformation [48]. Secondly, the slip of partial dislocation produces an HCP stacking sequence at the twin boundary to form a nanotwin–HCP lamella structure, and the HCP laths have a coherent relationship with the matrix. In general, phase transformation occurs at high stress levels, and HCP is beneficial to the energy reduction of CrCoNi alloy during large-strain and low-temperature stretching [40]. A few HCP sequences first appeared at the twin boundary at 50 μm depth, as shown in Figure 8b, and then multi-layer HCP laths were generated in the nanocrystalline structure, shown in Figure 9c, at a higher stress level. However, this transition was relatively minor. Thirdly, dislocation slip and pile-up destroy grain boundaries and the orientation relationship between twins or sub-grains and matrix to separate grains. On the one hand, dislocations slip to the twin boundary and interact with the twin, leading to a deviation in the orientation between the twin and the matrix and the formation of sub-grain boundaries. On the other hand, the accumulation of dislocations forms a dislocation band under high stress levels, which separates the grains and gradually forms a sub-grain boundary. Then, the low-angle boundary can evolve into a high-angle one upon further plastic strain.

The evolution of deformation twins in the deformation process of CrCoNi MEA varies with the strain level. The deformation twins begin to form at 150 μm depth in large quantities and maintain a high-density structure at each deformation stage. Several studies have found that when a dislocation slips across the twin boundary, mobile dislocation, pinned dislocation, and stacking faults occur at the twin boundary, complicating the twin boundary structure [49,50]. At the depth of 50~150 μm, the interaction between dislocations and deformation twins is so severe that the original coherent twin boundary loses its coherency with the matrix, as shown in Figure 8. When the dislocation accumulation at the twin boundary reaches a critical threshold, the dislocation will then cross the twin boundary and break the twin grain, contributing to the refinement of grains [51]. In the layer near the surface, the generation of twins requires greater critical stress due to the smaller grain size. The interaction between dislocations and twins slows down, resulting in a less complex twin boundary, which can be seen in Figure 9b. It is worth noting that the cross twins were only observed in coarse grains in this work. The grain size has an effect on the formation of twins. When the primary twin/matrix lamellae are extremely thin, the emission of dislocations from the primary twins is difficult, and thus the secondary twin cannot be generated in the narrow lamellae [26,34].

#### 3.3.2. The Nanocrystallization Formation Mechanism

**Figure 10:**
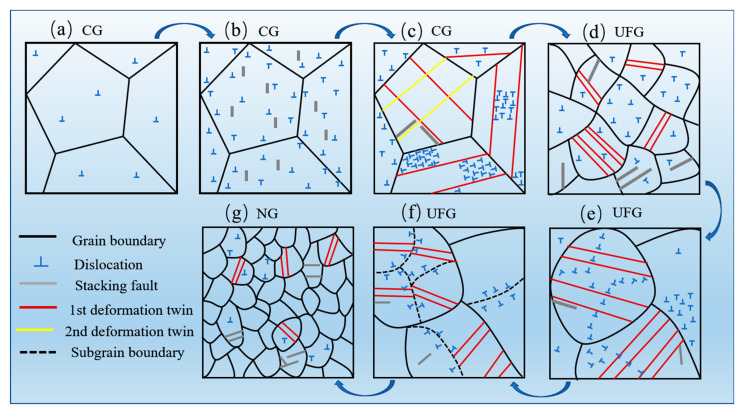
Schematic illustration of nanocrystallization mechanism of GNS CrCoNi MEA.

The nanocrystallization formation mechanism of CrCoNi MEA induced by HESP is schematically illustrated in Figure 10. The as-received alloy has a strain-free, coarse-grained structure with sporadic dislocation lines, as seen in Figure 10a. During the coarse grain deformation stage, the grain maintains its original shape and size. Dislocation tangles begin to form immediately after the multiple-slip system is actuated in grains, shown in Figure 10b, differing from the dislocation cell structure produced in high-stacking-fault-energy alloys under low stress, including Fe (200 mJ/m^2^) [27] and Ni (128 mJ/m^2^) [30]. As the stress level gradually increases, one or two twinning systems are activated within each grain, and interactions between dislocations and twins, as well as between twins themselves, occur, leading to the subdivision of coarse grains, as shown in Figure 10c. The dislocation bands separate the twin–matrix lamellae into ultrafine segments. The twin–twin interaction results in rhomboid blocks with high misorientation. Subsequently, these sub-segments transform into random ultrafine grains through dislocation slip or sub-grain rotation, seen in Figure 10d.

At the stage of ultrafine grain deformation, the dislocation–twin interactions dominate the deformation process. Figure 10e,f illustrate that a large number of dislocations accumulate around the twins, causing significant lattice distortion and breaking the coherent relationship between the twins and the matrix. Consequently, the coherent relationship between the twins and matrix is broken into incoherent HAGB during dislocation slip. Simultaneously, the dislocation bands also emerge, dividing the grains into elongated segments. In the nanograin deformation stage, a homogeneous and random nanograin structure is obtained, as presented in Figure 10g. The nanocrystalline structure will be continuously deformed into smaller parts due to the dislocation–twin interaction.

## 4. Conclusions

(1) A gradient nanostructure was formed on the surface of the CrCoNi MEA, consisting of distinct zones: a nanocrystalline zone, an ultrafine grain zone, a coarse-grained deformation layer, and a strain-free coarse-grained zone. The nanocrystalline layer had a thickness of approximately 20 μm, with the grain size refined to approximately 50 nm.

(2) The morphology of dislocations in the grains evolved from low-density dislocation lines to high-density dislocation entanglements and dislocation bands, progressing from the core to the surface. Dislocation activity resulted in the generation of stacking faults, deformation twins, and HCP structures. The deformation twins penetrated the grains, contributing to grain refinement, and the spacing between twin lamellae decreased with the increasing strain level.

(3) The nanocrystallization formation mechanism could be summarized as follows: the dislocation–twin/matrix and twin–twin interactions separate coarse grains into ultrafine grains. With further deformation, the severe interaction between the dislocation and twin breaks the coherent twin area, and highly misoriented HAGB is generated by sub-grain rotation. At a higher stress level, the stepped deformation twins refine the nanocrystalline structure to a smaller scale.

## Data Availability

The data are provided within the article.
